# Obstructive sleep apnea syndrome: aeronautical medical assessment

**DOI:** 10.47626/1679-4435-2024-1326

**Published:** 2025-09-22

**Authors:** José de Sousa Vale, Silvia Maria Pimenta

**Affiliations:** 1 Natividade Family Health Unit, Amadora-Sintra Local Health Unit, Lisbon, Portugal; 2 Medical Department, Hi Fly, Lisbon, Portugal

**Keywords:** accidents, sleep apnea, obstructive, aviation, aerospace medicine, occupational medicine., acidentes, apneia obstrutiva do sono, aviação, medicina aeroespacial, medicina do trabalho.

## Abstract

Obstructive sleep apnea syndrome is a highly prevalent condition. It falls under sleep-related breathing disorders, is linked to increased morbidity and mortality, and is believed to be markedly underdiagnosed. Practical guidelines for this syndrome in aeronautical/space occupational medicine remain scarce. According to the European Union Aviation Safety Agency, candidates with inadequately treated obstructive sleep apnea syndrome must be considered unfit. The Aerospace Medical Association encourages initiatives to raise awareness and ensure treatment, as it impacts pilot performance and flight safety. Thus, developing clinical guidelines targeting high-risk groups, addressing diagnosis, treatment, and monitoring, is essential. This study aims to guide attending physicians managing aeronautical/space industry workers, as well as roles requiring high attention and human-life responsibility, thereby minimizing risks linked to obstructive sleep apnea syndrome. Results obtained through narrative literature review.

## Abstract (pt)

A síndrome da apneia obstrutiva do sono é uma afecção muito prevalente. Enquadra-se nos distúrbios respiratórios do sono, associa-se ao aumento da morbimortalidade e estima-se que haja elevado subdiagnóstico. Existem poucas orientações práticas sobre essa síndrome na medicina do trabalho da área aeronáutica/espacial. A Agência Europeia para a Segurança da Aviação limita-se a referir que os candidatos com síndrome da apneia obstrutiva do sono tratada de forma insatisfatória devem ser considerados inaptos. A Aerospace Medical Association incentiva quaisquer iniciativas que aumentem a sua percepção e levem ao seu tratamento, uma vez que isso afeta o desempenho dos pilotos e a segurança do voo. Por isso, é importante estabelecer orientações clínicas voltadas aos grupos de maior risco de acidentes, abordando diagnóstico, tratamento e acompanhamento adequados. Este estudo tem como objetivo orientar os médicos assistentes de trabalhadores da indústria aeronáutica/espacial, bem como de funções que exijam altos níveis de atenção e responsabilidade pela vida humana. Dessa forma, os riscos associados à síndrome da apneia obstrutiva do sono poderão ser minimizados. Para a obtenção dos resultados, utilizou-se uma revisão narrativa da literatura.

## INTRODUCTION

Obstructive sleep apnea syndrome (OSAS) is a sleep-related breathing disorder marked by significant airflow interruption or reduction despite respiratory effort. During sleep, upper airway resistance increases and functional residual capacity declines, promoting upper airway obstruction and reduced inspiratory airflow.^1^ The American Academy of Sleep Medicine (AASM) defines OSAS as repetitive episodes of upper airway obstruction during sleep, typically associated with oxyhemoglobin desaturations.^2^

More than 1 billion individuals worldwide suffer sleep-related breathing disorders, being OSAS the most common form.^3^ Prevalence of OSAS is about 23.4% in women and 49.7% in men.^4^ Estimates indicate higher incidence in women from age 65, rising post-menopause, while in men peak incidence occurs between 45 and 64 years.^1^ Community-based studies show a male-to-female ratio of 2:1 to 4:1.^1^

Healthcare costs associated with OSAS are 3 times higher than those for individuals without the condition, yet over 80% of cases remain undiagnosed.^5^ OSAS is associated with significant adverse behavioral and physical effects. Behavioral changes include excessive daytime sleepiness, reduced attention, difficulty concentrating, mood disturbances and neuropsychological dysfunction. Physical consequences include cardiovascular alterations, mainly hypertension, arrhythmias and increased risk of ischemic events.^6^,^7^

Risk of hypertension is 3 times higher, as is that for coronary artery disease (1.3x) and stroke (1.6x).^8^ OSAS is also linked to insulin resistance and diabetes mellitus.^9^ Neuropsychological changes, notably daytime sleepiness, increase risk of traffic accidents (8 times higher).^6^ Cost of untreated OSAS, compared with treated cases, is 2 to 3 times higher, mainly due to increased risk of cardiovascular disease and traffic accidents.^6^

OSAS increases traffic and occupational accident risk by approximately 2 to 7-fold.^6^ Adequate treatment is known to substantially reduce accident risk.^10^ Accident rates linked to OSAS carry significant legal implications and raise questions regarding examinations for license/certification renewal and maintenance in patients with the syndrome.^10^ To minimize OSAS impact, early identification of at-risk patients is essential for effective treatment.

This study aims to guide attending physicians caring for aviation-industry workers, given scarce scientific literature for this population. To that end, this article employs a narrative literature review to outline the concept of OSAS, its diagnostic and therapeutic criteria, and proposes a specialized aeromedical evaluation protocol based on available literature.

## METHODS

Three databases (PubMed, Cochrane, Google Scholar) were searched with this query: “OSAS” AND “Adult” AND “Diagnosis” AND “Treatment” OR “Pilot” AND “OSAS” OR “Cabin Crew” AND “OSAS” OR “Accident” AND “OSAS.” The search yielded 404 results. Reviews, meta-analyses, and systematic reviews on these topics, plus articles published between 2000 and 2023, were prioritized. Analysis was limited to studies in English or Portuguese. Titles meeting search criteria were screened by abstract and, when relevant, by full text.

Excluded articles were those: published before 2000; incomplete; not available in English or Portuguese; duplicates; and not focused on relevant topics. Literature search identified 59 articles, 27 excluded for failing inclusion criteria or providing insufficient abstract details. A total of 32 articles were included and reviewed. A narrative literature review was employed to synthesize findings.

## RESULTS

### DIAGNOSIS

Given that this syndrome is heterogeneous, with multiple predisposing factors, pathophysiological mechanisms, clinical manifestations and consequences from respiratory events, OSAS poses challenges for recognition in clinical practice.^11^ Evidence shows substantial differences in OSAS symptoms, diagnosis and outcomes between men and women.^12^ Several authors propose the existence of OSAS phenotypes, expected to enable more precise diagnosis and treatment strategies.^13^

Personal history and physical examination, including blood pressure and body mass index (BMI) measurement, predict OSAS presence in about 50% of patients.^14^ Evaluation should address snoring, witnessed apneas, episodes of gasping or choking during sleep, restless sleep and excessive daytime sleepiness. Work performance, driving difficulty and any prior traffic accidents related to daytime sleepiness should also be assessed.

Evaluation should also address morning headaches, xerostomia, alcohol intake, weight gain and mood changes. A complete personal and family history, including use of medications such as narcotics, muscle relaxants and sedatives, is essential. Physical examination should include assessment of weight, neck circumference, craniofacial anomalies, oropharyngeal findings and presence of other risk factors or cardiovascular disease.^2^

Thyroid function tests should be performed if thyroid dysfunction is suspected. Fasting glucose measurement is recommended, as OSAS independently increases risk of developing diabetes mellitus. Complete blood count with iron kinetics study is useful in suspected anemia, as is ferritin level in suspected restless legs syndrome. Spirometry with bronchodilator testing is indicated if pulmonary disease is suspected, and 12-lead electrocardiogram or echocardiogram is indicated if cardiac pathology is suspected.^2^

Polysomnography (PSG) is gold-standard test for OSAS diagnosis.^2^ This test reveals hypoxemia/desaturation severity, sleep fragmentation, OSAS-associated cardiac arrhythmias, and apnea/hypopnea events. For clinical significance, apnea and hypopnea events must last at least 10 seconds, occur 10 to 15 times per hour, and cause oxygen saturation decrease or sleep arousals.^2^

Severity of OSAS is determined by the Apnea-Hypopnea Index (AHI) (Chart 1), calculated by dividing total apneas and hypopneas by total sleep time. Diagnosis is established when AHI ≥ 5 with associated symptoms, or AHI ≥ 15 regardless of symptoms. Criteria for mild OSAS require symptoms such as excessive daytime sleepiness, cardiovascular disease, hypertension and mood changes. PSG should be performed during patient’s typical sleep hours and include all sleep stages in supine position. However, it has long been recognized that AHI alone fails to capture OSAS patient heterogeneity.^13^

**Chart 1:** Severity and diagnostic metrics ^2^

PSG level 1 (laboratory)	
AHI	Normal: < 5 episodes/hour
	Mild sleep apnea: ≥ 5 and < 15 episodes/hour, with symptoms
	Moderate sleep apnea: ≥ 15 and < 30 episodes/hour
	Severe sleep apnea: ≥ 30 episodes/hour
RDI	≥ 5 and < 15 episodes/hour, with symptoms

PSG nível 2 (ambulatório)	
REI	≥ 15 episodes/hour

AHI = Apnea Hypopnea Index; PSG = polysomnography; RDI = Respiratory Disorders Index; REI = Respiratory Events Index.

PSG can be performed in laboratory (level 1) or ambulatory (level 2) settings, with level 1 involving overnight monitoring (typically by a technician) and video recording during sleep. Cardiorespiratory monitoring without electroencephalography, electro-oculography or electromyography may be used in cases of high OSAS suspicion. Laboratory PSG is recommended for OSAS diagnosis in patients with significant cardiorespiratory disease, potential neuromuscular pathology, hypoventilation during wakefulness or sleep, chronic opioid use, history of cerebrovascular accident, severe insomnia, and suspected behavioral disturbances during rapid eye movement (REM) sleep and non-REM sleep.^2^

In split-night PSG, compared with full-night PSG, the study is conducted in two phases. In the first part of the night, patients are diagnosed with OSAS; in the second, titration of positive airway pressure (PAP) begins. Split-night PSG allows initiation of continuous positive airway pressure (CPAP) titration when at least 3 hours of sleep remain, provided moderate to severe OSAS is observed for a minimum of 2 hours of recording.^15^ However, it has drawbacks, including limited assessment of REM sleep, reduced evaluation time in patients with difficulty initiating sleep, and insufficient time to determine appropriate CPAP settings.^15^

In clinical practice, the Epworth Sleepiness Scale (ESS) is routinely used for diagnosing and monitoring treatment efficacy for daytime symptoms.^16^ ESS assesses the likelihood of dozing in 8 situations over the past 30 days. It is probably the most used questionnaire in sleep medicine. However, it shows weak correlation with objective sleepiness measures, and its items are prone to misinterpretation.^17^

In patients with suggestive complaints, several algorithms have been developed over the years to more accurately identify suspected OSAS. These combine clinical variables such as BMI, neck circumference, mandibular structure, snoring, witnessed nocturnal respiratory disturbances and hypertension. Although sensitivity can be high (78-95%), specificity remains low (41-63%).^18^

Berlin Questionnaire and STOP-Bang are screening tools widely used and validated. In the STOP-Bang test, risk is calculated by summing “yes” responses across 8 parameters; each “yes” equals 1 point, yielding a total score from 0 to 8. OSAS risk is low when score < 3; moderate when score ≥ 3 and ≤ 4; and high when score ≥ 5.^19^

According to Chung et al.,^19^ a STOP-Bang cutoff of 4 provides better balance between specificity and sensitivity compared with a cutoff of 3 in obese populations. In the improved STOP-Bang version described by Chung et al. in 2016,^19^ patients initially classified with intermediate OSAS risk can be re-stratified if they test positive on at least one of the following parameters: BMI > 35 kg/m^2^, neck circumference > 43 cm in men and > 41 cm in women. However, the risk assessment tool validated for the Portuguese population uses a cutoff of 3.^19^

The Berlin Questionnaire comprises 10 items on snoring, unrefreshing sleep, drowsiness while driving, sleep apneas, hypertension and BMI.^20^ Results stratify patients as high or low OSAS risk. High-risk score on Berlin Questionnaire is associated with sensitivity 80% and specificity 46% when OSAS is defined as AHI 5-14 events/hour, and sensitivity 91% and specificity 37% when OSAS is defined as AHI ≥ 15 events/hour.^20^

Questionnaires and algorithms are not recommended for OSAS diagnosis, even when polysomnography cannot be performed.^17^,^20^

### TREATMENT

Adult OSAS treatment continues to evolve, and lifestyle modification is most frequently recommended. Weight loss reduces symptom severity in obese patients and, if significant, may allow some to discontinue CPAP therapy. Weight loss via bariatric surgery may improve OSAS; however, bariatric surgery vs. conventional weight-loss therapy does not significantly reduce AHI, despite large weight-loss differences. Lifestyle modifications should be considered adjuvant, not curative, therapy,^21^ and lack of symptom improvement should not prevent patients from initiating CPAP treatment.^17^

The most effective therapy for mild, moderate or severe OSAS is nocturnal non-invasive ventilation - CPAP.^22^ CPAP treatment uses mask interface and flow generator to prevent airway collapse, thereby preventing apnea, hypoxia and sleep disturbances. CPAP significantly improves objective measures such as 24-hour systolic and diastolic blood pressure, and subjective measures such as ESS scores in OSAS patients with daytime sleepiness. CPAP may also reduce atherosclerosis risk and improve insulin resistance in non-diabetic patients.

There are various CPAP interface types, including nasal masks, oral masks and nasal pillows. In patients with poor adherence, alternative interface should be considered.^22^ Other positive-pressure methods include bilevel positive airway pressure (BiPAP), delivering predetermined inspiratory and expiratory pressures, and automatic positive airway pressure (APAP), which adjusts pressure in response to airflow changes, snoring or circuit pressure.^2^

Oral appliances are available for OSAS treatment, often preferred by patients, such as mandibular advancement devices (MADs) and tongue-retaining devices. Although CPAP is more effective than MADs at reducing AHI (4.5 vs. 11 events/hour, respectively), adherence to MADs is higher (6.5 vs. 5.2 hours/night).^23^ No statistically significant differences have been observed between MADs and CPAP in terms of blood pressure reduction, daytime sleepiness or quality of life.^24^

If OSAS occurs only in the supine position, changing sleep position may be effective.^2^ Symptoms of excessive daytime sleepiness that persist despite good adherence to CPAP or other therapies may warrant additional investigation.^2^

Patients should be considered for surgery if multiple CPAP attempts fail and oral appliance use is not feasible, since surgery may improve adherence. Surgical indication must be based on airway collapse site. Nasal septoplasty/turbinectomy should be offered to patients with nasal septal deformities. Uvulopalatopharyngoplasty, which resects uvula and soft palate, benefits only a limited subset of patients. Predicting which patients will respond to this intervention remains difficult.

Additional surgical options include insertion of palatal implants and osteogenic distraction for maxillary expansion in high-arched palates. Hypoglossal nerve stimulation, via surgical implantation of a device in the upper chest, assists recruitment of tongue muscles, reduces pharyngeal collapsibility, and decreases upper-airway resistance. Tracheostomy is generally reserved for very severe OSAS unresponsive to medical therapy or presenting cor pulmonale.^2^

According to Portuguese clinical guideline, once home PAP device use is initiated, patient training and education should begin. All OSAS patients on CPAP or BiPAP are monitored for adherence and efficacy. Adherence is defined as use > 4 hours on more than 70% of nights.^25^ Therapeutic efficacy is defined as AHI < 5.^25^ Excessive daytime sleepiness should be assessed using ESS. Patients should be re-evaluated 1-3 months after treatment initiation, then every 6 months, and, if well controlled, annually.^25^

### AEROMEDICAL/OCCUPATIONAL HEALTH ASSESSMENT

OSAS should be suspected when patient reports daytime sleepiness, snoring, witnessed apneas or morning headaches, particularly in presence of risk factors such as obesity, male sex and advanced age.

Use of screening questionnaires for OSAS risk is not recommended, as none has proven superior to clinical history and physical examination;^26^ however, they may be useful in certain contexts.^20^ The most commonly used screening tools include STOP-Bang, ESS and the Berlin Questionnaire.^27^

Some aviation-industry stakeholders have expressed concern regarding OSAS screening, asserting it is an urgent flight-safety issue.^28^,^29^ The Easy Access Rules for Aircrew (EASA), in its latest recommendations, do not address sleep disorder screening, instead merely stating candidates with inadequately treated OSAS must be deemed unfit.^30^

The Federal Aviation Administration (FAA) recommends OSAS risk assessment using integrated evaluation of history, symptoms and clinical findings, following AASM guidelines. In high-risk patients, OSAS evaluation should be completed within 90 days, with PSG as indicated.^28^

According to the UK Civil Aviation Authority,^31^ if aircrew comply with ventilatory therapy, CPAP must be used for at least 5 hours per night, 6 nights per week. Machine usage report and logbook should be reviewed every 3 months during the first year of treatment.^31^ Pilots must not fly if adherence is poor, symptoms recur, or ESS score is ≥ 10.^31^

According to FAA,^32^ the attending physician should grant authorization if the candidate reports symptoms severe enough to pose an immediate flight-safety risk. The PAP device report must cover 30 days of uninterrupted therapy for new diagnoses (a minimum of 2 weeks may be acceptable if data demonstrate excellent adherence, effective treatment and absence of symptoms), or 365 days for previously diagnosed and treated candidates. Medical authorization requires PAP adherence on 75% of days, with at least 6 hours’ use per night, and an AHI < 5.^32^

According to the existing literature, upon OSAS suspicion an integrated evaluation of history, symptoms and clinical findings should follow AASM guidelines.^32^ risk-assessment tools such as STOP-Bang, ESS or the Berlin Questionnaire may be applied. In cases of increased risk, temporary unfitness and level 1 or 2 PSG for diagnostic confirmation are recommended. OSAS evaluation should be completed within 90 days.^32^

If treatment is instituted, adherence criteria include ESS score < 10,^31^ PAP use on at least 75% of days with a minimum of 6 hours per night, and AHI < 5.^32^ For newly diagnosed patients on PAP, the report must cover 30 consecutive days of uninterrupted use.^32^ Subsequent reassessment should occur every 3 months during the first year,^31^ and if well controlled, semiannually.^31^ During this period, certification should be issued with limitations.

**Figure 1:**
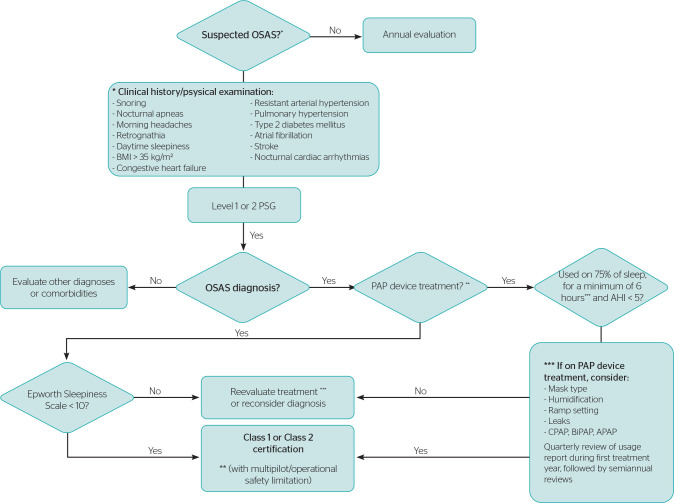
Aeromedical evaluation flowchart in obstructive sleep apnea syndrome (OSAS). APAP = automatic positive airway pressure; BiPAP = bilevel positive airway pressure; CPAP = continuous positive airway pressure; BMI = body mass index; PAP = positive air pressure; AHI = Apnea-Hypopnea Index.

Figure 1 presents a suggested decision flowchart for aeromedical evaluation, taking into account the existing literature.

## DISCUSSION

OSAS is a prevalent, highly heterogeneous sleep condition - despite lacking formal characterization as such - which leads to underdiagnosis. It is imperative that operational personnel obtain the necessary amount of sleep in their daily routine. Although aviation regulators enforce duty and rest schedules, when work-related sleep interruptions are compounded by fragmentations associated with medical pathologies, the resulting additive effects can be severe.

Untreated OSAS is considered a disqualifying medical condition. Pilots with OSAS may continue flying provided there is adequate follow-up and adherence to treatment. The FAA advises clinicians to remain vigilant for OSAS risk and other sleep disorders. EASA is silent on both this issue and on certification of crew with treated OSAS.

This study aims to guide occupational and aviation health physicians in the suspicion and diagnosis of OSAS, seeking to minimize risks and preserve both flight safety and the performance of aviation professionals.

Ultimately, despite scientific advances in diagnostic methods and personalized treatments, OSAS still receives less attention than other sleep-related respiratory disorders, and the scientific community must concentrate efforts to reverse this trend.
